# The floating hip injury: a descriptive study and case-control analysis

**DOI:** 10.1177/11207000231160075

**Published:** 2023-03-13

**Authors:** Mark Y Z Wong, Marios Ghobrial, Win M Han, Joseph Alsousou, Andrew Carrothers, Peter Hull, Daud Chou, Jaikirty Rawal

**Affiliations:** 1 Cambridge Orthopaedic Pelvic Unit (COPU), Department of Trauma and Orthopaedic Surgery, Addenbrooke’s Hospital, Cambridge University Hospitals NHS Foundation Trust, Cambridge, UK; 2 University of Cambridge School of Clinical Medicine, Cambridge, UK

**Keywords:** Acetabular fracture, femur fracture, floating hip, ipsilateral, pelvic fracture

## Abstract

**Purpose:**

A “floating hip” (FH) injury is a rare injury describing the simultaneous ipsilateral fracture of the femur and pelvis or acetabulum (P/A). We describe our experience with patients presenting with FH injuries and compare them to controls with similar P/A fractures but without femoral involvement.

**Methods:**

Medical records and radiographs of FH patients and controls presenting to our tertiary centre between 2015 and 2020 were reviewed. Follow-up data from outpatient clinical records were also extracted. The control group were extensively matched by age, sex, body mass index, fracture classification and energy of injury.

**Results:**

From 1392 recorded P/A fractures, 42 FH cases were identified (average age 39 years, 78.6% males). The most common femoral fracture was the midshaft (35.7%), followed by the neck of femur (26.2%). 90.5% of FH injuries were due to high-energy mechanisms. 64.3% of P/A fractures, and 100% of femoral fractures were managed surgically. Compared to controls, FH cases were more likely to have additional orthopaedic injuries (73.8% vs. 40.5%, *p* *=* 0.002), more total theatre admissions (mean 2.5 vs. 1.19, *p* *<* 0.001), longer hospital stays (28.3 vs. 14.9 days, *p* *=* 0.02), and a higher rates of post-op complications (53.8% vs. 20%, *p* *=* 0.025)

**Conclusions:**

We report differences in the presentation, management, and outcomes of FH injuries versus controls, even after extensive matching for confounders. These differences may inform future treatment strategies for the FH injury.

## Introduction

The “floating hip” (FH) is an uncommon injury describing the simultaneous ipsilateral fracture of the femur and pelvis or acetabulum (P/A), resulting in fractures both proximal and distal to the hip joint.^1,2^ A simplified 3-type classification has been proposed, Type A referring to a fracture involving the acetabulum, Type B involving the pelvis and Type C involving both pelvic and acetabulum.^3^ High-energy trauma pathomechanisms often underlie FH injuries, and such injuries are thus often associated with additional injuries and complications.^4–6^ This combination of fractures in conjunction with associated injuries, therefore, pose crucial implications for the management of such patients.^7,8^

Despite its existence in literature for over 20 years, disagreement still exists over whether the “floating hip” should be considered a distinct injury.^4,5,8–11^ Evidence remains equivocal in particular, on whether FH injuries are associated with unique complications akin to the more established floating knee or floating elbow injuries.^5,8,9^ A growing number of studies have described their encounters with this rare injury.^5,10–13^ However, many studies to date have either only reported case-series of limited numbers, or have offered no form of controlled comparison to similar P/A injuries without femoral involvement.

This paper reviews our experience with this injury at a Level 1 trauma centre, and provides a description on the injury patterns, management, and outcomes of such patients presenting with the FH injury. This paper then compares the results of these FH cases to those of case-matched patients presenting with similar pelvic or acetabular fractures without ipsilateral femoral involvement. These findings will offer insight into whether there are typical differences both in presentation and clinical outcomes in FH cases as compared to selected controls and may further inform whether these differences should warrant an impact on treatment strategies for the FH injury.

## Methods

### Design and study population

This is a retrospective matched case-control study of prospectively collected data. The inclusion criteria for FH cases were as follows: patients of any age, presenting with an ipsilateral P/A fracture and a femoral fracture. Both conservatively- and surgically-managed cases were included. Exclusion criteria included patients with contralateral P/A and femoral fractures, patients where the P/A and femoral fractures did not occur simultaneously, and pathological fractures due to malignancies.

Our prospectively collected database was searched for patients fulfilling the FH injury criteria. Between the years 2015 and 2020, 1392 patients sustaining either a pelvic or acetabular fracture were referred to our Level 1 major trauma centre in the United Kingdom. Of these 1392 patients, 57 presented with concurrent femoral fractures, of which 50 were ipsilateral to the pelvic or acetabular fracture and fit our inclusion criteria. We further excluded 2 patients with isolated iliac wing fractures as such fractures did not affect the integrity of the pelvic ring. Lastly, 6 patients with Pipkin IV fractures (fractures of the femoral head with a concomitant acetabular fracture) were excluded as these fractures were deemed a distinct fracture pattern of their own. A total of 42 patients thus formed our FH case-series.

### Data collection and studied parameters

Medical records and radiographs were retrospectively reviewed by independent clinicians not involved in the care of the patient. Patient demographics, injury details, fracture classifications, management and postoperative outcomes were extracted. Any discrepancies were referred to the senior authors.

Patient demographics included age, sex and body mass index (BMI). Injury mechanisms were stratified into 2 groups: (1) high-energy trauma (road traffic accidents [RTA], crush injuries, falls from height ⩾6 feet); and (2) low-energy trauma (simple falls, falls from height ⩽6 feet). Associated injuries were divided into (1) other orthopaedic injuries (not including a pelvic, acetabular or femoral fracture), and (2) non-orthopaedic injuries. Management options were divided into surgical and conservative, with further surgery-related details including type of procedure, method of fixation, time to theatre, and time in theatre, extracted when relevant. Weight-bearing status was defined as per surgeon recommendation post-op. Complications and post-surgical outcomes were obtained from follow-up outpatient clinic records and radiographs when available, and included pulmonary embolisms (PE), infections (superficial or deep), post-traumatic osteoarthritis (PTO), heterotopic ossification (HO), avascular necrosis (AVN), fracture malunions and the need for additional readmission or revision surgery.

#### Selection of control group

**Figure 1.**
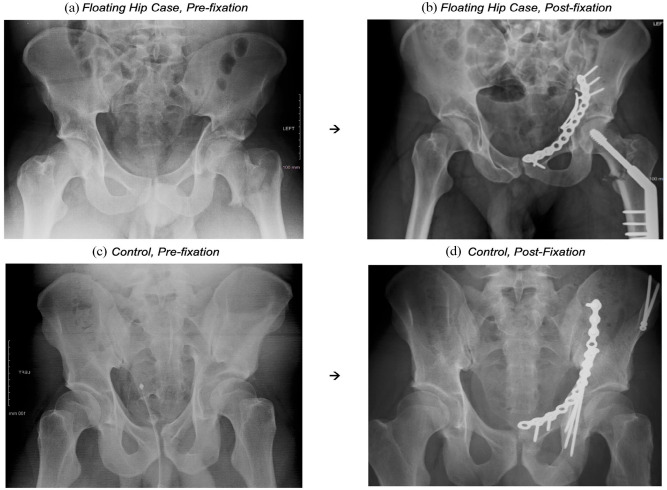
Classical “floating hip” injury presentation (and associated control): (a) “floating hip”, 23 Male, high-energy impact fall from height, L NOF + L ABC acetabular fracture, (b) acetabular fracture managed by ORIF (plate fixation), NOF fracture managed by DHS, (c) control, 28 Male, high-energy impact from RTA, R ABC acetabular fracture, and (d) managed by ORIF (plate fixation + screws). Arrows (→) depict the same patient, before and after operative fixation.

The control group comprised of 42 different patients who presented with either a pelvic or acetabular fracture, but without an ipsilateral femoral fracture. These patients were drawn from the same database of 1392 patients during the same study period. To enable optimal comparisons of management and postoperative outcomes across both groups, controls were matched by age (±5 years), sex, fracture classification, mechanism of injury (High vs. Low), and BMI (±2). For FH cases with combined P&A fractures, controls were matched to the fracture that was operatively managed. Figure 1 offers an illustration of a FH injury ((A) pre-surgery and (B) post-surgery), alongside an associated control.

### Fracture classifications

Acetabular fractures were classified on radiographs and computer tomography scans according to the established Judet and Letournel classification for acetabular fractures, while Pelvic fractures were classified according to the Young-Burgess classification.^14,15^ The classifications of P/A fractures in both cases and controls are detailed in Supplemental Material Table 1. Femoral fractures were grouped by location: neck of femur (NOF), trochanteric, proximal shaft, midshaft and distal. These were further grouped into proximal (NOF + trochanteric + proximal shaft), middle and distal femoral fractures.

Combinations of P/A and femoral injuries were grouped according to the original Liebergall FH classification as detailed earlier, into Type A (acetabular + femur), Type B (pelvis + femur) and Type C (combined P&A + femur). Within Type A FH injuries, acetabular fractures were further grouped into either posterior (any fracture involving the posterior column or posterior wall) or central (double column involvement, or transverse fractures without posterior involvement) as detailed in Liebergall et al.^3,8^ and subsequent studies.

### Statistical analysis

Differences in categorical values (between FH cases and controls) were compared using the *X*^2^ statistic for proportions or the Fischer exact test when appropriate. Differences in continuous variables were evaluated using either an independent sample *t*-test or a Mann-Whitney U-test for non-parametric data. A *p-*value of <0.05 was considered statistically significant. All statistical analyses were performed using Stata 14.1 (StataCorp, College Station, TX, USA).

## Results

### Demographics, fracture classifications and injury mechanisms of floating hip injuries

A total of 42 patients with FH injuries were identified, mean age 39.0 (range: 15–86) years, of which 33 (78.6%) were male. 13 (31.0%) had Type A FH fractures, 23 (54.8%) had Type B FH fractures while 6 (14.3%) had Type C FH fractures. The most common site of femoral fracture was at the midshaft (35.7%), followed by the NOF (26.2%). Table 1 provides a detailed breakdown of the FH fracture types.

**Table 1.** Combination patterns of acetabular/pelvis fracture and femoral fracture in patients with floating hip injuries.

	Femur fracture site			Total, *n* (%)
	Proximal	Mid	Distal	
Acetabular fractures (Type A)				
Total acetabular, *n* (%)	7 (53.8)	5 (38.5)	1 (7.69)	13 (30.9)
Posterior, *n* (%)	3 (50.0)	2 (33.3)	1 (16.7)	6 (14.3)
Central, *n* (%)	4 (57.1)	3 (42.9)	0 (0.0)	7 (16.7)
Pelvic fractures (Type B)				
Total pelvic, *n* (%)	14 (60.1)	6 (26.1)	3 (13.0)	23 (54.8)
APC type, *n* (%)	4 (40.0)	4 (40.0)	2 (20.0)	10 (23.8)
LC type, *n* (%)	10 (76.9)	2 (15.4)	1 (7.7)	13 (31.0)
Combined fractures (Type C)				
Total combined, *n* (%)	1 (16.7)	4 (66.7)	1 (16.7)	6 (14.3)
APC + central, *n* (%)	1 (33.3)	1 (33.3)	1 (33.3)	3 (7.1)
LC + central, *n* (%)	0 (0.0)	2 (100)	0 (0.0)	2 (4.8)
SIJ disruption + posterior, *n* (%)	0 (0.0)	1 (100)	0 (0.0)	1 (2.4)
Total, *n* (%)	22 (52.4)	15 (35.7)	5 (11.9)	42

The majority of FH injuries (90.5%) were due to high-energy trauma pathomechanisms. The injury mechanisms were largely due to RTA (64.3%), with the remaining mechanisms detailed in Table 2. The majority of FH cases presented with other orthopaedic injuries (81.0%) as well as other non-orthopaedic injuries (73.8%).

**Table 2.** Comparison of demographics, injury details and management between floating hip cases and control groups.

	Group		*p-*Value
	Cases (*N* = 42)	Controls (*N* = 42)	
	*n* (%)	*n* (%)	
Demographics and presentation			
Basic demographics			
Gender (Male)	33 (78.6)	33 (78.6)	1.00
Age (Mean ± SD)	39.0 (±3.18)	38.5 (±3.10)	0.91
BMI (Mean ± SD)	25.5 (±0.77)	24.3 (±0.82)	0.28
Injury mechanism (energy)			
High (vs. Low)	38 (90.5)	38 (90.5)	1.00
Mechanism subtypes			
RTA	27 (64.3)	31 (73.8)	0.55
Fall from height	6 (14.3)	5 (11.9)	
Fall	4 (9.52)	2 (4.76)	
Crush	2 (4.76)	0 (0.0)	
Others	3 (7.13)	4 (9.52)	
Fracture patterns			
Central acetabular	7 (16.7)	8 (19.1)	0.42
Posterior acetabular	6 (14.3)	7 (16.7)	
Pelvic APC	10 (23.8)	12 (28.6)	
Pelvic LC	13 (31.0)	14 (33.3)	
Combined	6 (14.3)	1 (2.38)	
Other injuries			
Other ortho injuries	34 (81.0)	23 (54.8)	**0.01**
Spine/vertebral	12 (28.6)	9 (21.4)	
Upper limb	20 (47.6)	9 (21.4)	
Hand	4 (9.52)	13 (31.0)	
Lower limb (excluding femur)	15 (35.7)	6 (14.3)	
Foot	5 (11.9)	0 (0.0)	
Other non-ortho injuries	25 (73.8)	26 (69.1)	0.82
Head	15 (35.7)	8 (19.1)	
Thorax	17 (40.4)	16 (38.1)	
Abdomen	10 (23.8)	10 (23.8)	
Pelvis	5 (11.9)	5 (11.9)	
Management			
Overall management			
Surgical management of P/A fracture	27 (64.3)	25 (59.5)	0.65
Conservative management of P/A fracture	15 (35.7)	17 (40.5)	
Surgical management of femoral fracture	42 (100.0)	NA	NA
P/A surgical management*			
ORIF alone	15 (55.6)	18 (72.0)	0.59
ORIF + perc fixation	5 (18.5)	3 (12.0)	
Acute THR	3 (11.1)	1 (4.00)	
Perc fixation	1 (3.70)	3 (12.0)	
INFIX	2 (7.41)	0 (0.0)	
Exfix	1 (3.70)	0 (0.0)	
Number of theatre admissions			
Total	2.5 (2.04)	1.19 (1.40)	**<0.001**
For definitive Mx of P/A and/or femur	1.40 (0.50)	0.60 (0.50)	**<0.001**
For revision surgery	0.48 (1.15)	0.14 (0.65)	**0.03**
For other ortho injuries	0.36 (0.66)	0.36 (0.76)	0.9
For other non-ortho injuries	0.31 (0.75)	0.12 (0.33)	0.13
Hospital parameters			
Time to P/A procedure (hrs)*	67.5 (±14.2)	68.7 (±10.9)	0.95
Time in theatre (mins)*	371 (±40.4)	278 (±28.7)	0.07
Time of procedure (mins)*	246 (±18.3)	203 (±25.2)	0.17
Hospital stay (days)	28.3 (±5.3)	14.9 (±2.31)	**0.02**
Weight-bearing status			
FWB	5 (11.9)	5 (11.9)	0.89
WBAT	8 (19.1)	5 (11.9)	
TTWB	6 (14.3)	5 (11.9)	
PWB	1 (2.38)	1 (2.38)	
NWB	22 (52.4)	26 (61.9)	

SD, standard deviation; RTA, road traffic accidents; ORIF, open reduction and internal fixation; THR, total hip replacement; INFIX, internal fixator; FWB, full weight-bearing; WBAT, weight bear as tolerated; TTWB, toe touch weight-bearing; PWB, partial weight-bearing; NWB, non-weight-bearing. Bolded p-values indicate statistically significant differences.

* Only patients who underwent surgical management were included in this analysis and formed the baseline.

### Management of floating hip injuries

27 (64.3%) of the FH cases required surgical management of their P/A fracture (Table 2). An open reduction and internal fixation (ORIF) alone was the most common surgical procedure (55.6%), followed by an ORIF + percutaneous fixation (19.5%). In contrast to the P/A fractures, all FH cases required surgical management of their femoral fracture, with ORIF (73.8%) the most common procedure, followed by closed reduction internal fixation, and an acute THR (both 7.14%). Table 3 details the specific surgical management of individual FH cases.

**Table 3.** Surgical management of individual floating hip injuries.

Info	Injury mechanism	Femoral fracture	Pelvic fracture	Acetabular fracture	Fixed first	P/A treatment	P/A -fixation method	P/A – surgical approach	P/A – patient position	Femoral treatment	Femur -fixation method	Femur -approach	Complications
m, 32	RTA	Distal	APC II	T	Femur	ORIF	Plate	Stoppa (Pelvis) + KL (Acetabulum)	Supine + lateral	ORIF	IM Nail	Retrograde	Revision required for femoral screw
f, 44	Fall from Height	Proximal/Proximal Shaft	LC II		Same Procedure, P/A	ORIF	Plate	Stoppa & Lateral Window	Supine	ORIF	LCP Plating Proximal Femur	Direct Lateral	Deep infection – washout required
m, 26	RTA	Midshaft	APC III		Femur	Perc fixation	Perc SI Screw	Percutaneous	Supine	ORIF	IM Nail	Retrograde	Femoral nonunion – revision required
m, 38	RTA	Midshaft		TPW	–	Conservative	NA	NA	NA	ORIF	IM Nail	Retrograde	Not recorded
m, 68	RTA	Distal	APC II		Femur	ORIF	Plate + Screws	Stoppa	Supine	ORIF	IM Nail	Retrograde	Femoral nonunion – revision required
f, 80	Fall	NOF		PC	Fix and replace	Fix and replace	Fix and replace	KL (fix and replace)	Lateral	Fix and replace	THR (fix and replace)		
m, 23	RTA	Midshaft		AW	–	Conservative	NA	NA	NA	ORIF	IM Nail	Antegrade	Fat embolism
m, 17	RTA	Shaft		ACPHT	Same Procedure, Femur	ORIF	Plate + Screws	Lateral Window	Supine	ORIF	IM Nail	Antegrade	
m, 33	RTA	Shaft		AC	Same Procedure, Femur	EUA	NA	NA	NA	ORIF	IM Nail	Antegrade	
m, 41	Crush Injury	Distal/MidShaft	APC III		Same Procedure, Femur	ORIF + Perc Fixation	Plate + Perc SI Screws	Stoppa	Supine	ORIF	IM Nail	Retrograde	Sciatic nerve injury and foot drop
f, 21	Crush Injury	Distal/Midshaft	LC I	AC	Femur	ORIF + Perc Fixation	Plate + Perc SI Screws	Stoppa	Supine	ORIF	IM Nail	Retrograde	
m, 23	Other	NOF		ABC	Same Procedure, Femur	ORIF	Plate	Stoppa	Supine	Closed Reduction Internal Fixation	DHS	Lateral	Femoral nonunion – revision required
m, 27	RTA	Subtrochanteric	LC I		–	Conservative	NA	NA	NA	Exfix, ORIF	IM Nail	Antegrade	Pneumothorax
m, 41	Fall from Height	Shaft	LC III	ABC	P/A	ORIF + Perc Fixation	Screws + Perc SI Screws	Percutaneous	Supine	ORIF (L), Ex Fix (right)	IM Nail (L), Ex-fix^®^	Retrograde	PE
m, 53	RTA	Distal Shaft	APC I		–	Conservative	NA	NA	NA	ORIF	IM Nail + Poller Screws	Retrograde	Foot drop/anaesthesia
f, 63	RTA	NOF	LC I		Fix and replace	Fix and replace	Fix and replace	KL (fix and replace)	Lateral	Fix and replace	THR (fix & replace) + perc SI screws		
m, 57	RTA	Distal	LC I		Same procedure, femur	EUA	NA	NA	NA	ORIF	Plate (L), IM Nail I	Retrograde	Femoral nonuni–n - revision required
m, 19	RTA	Midshaft	APC I		–	Conservative	NA	NA	NA	ORIF	IM Nail	Retrograde	
m, 27	Fall from Height	Proximal/proximal shaft	LC I		Same Procedure, Femur	Perc Fixation	Perc SI Screws	Percutaneous	Supine	ORIF	IM Nail	Antegrade	
m, 35	Other	NOF + midshaft	APC III	Tx	Femur	ORIF + Perc Fixation	Plate + Screws + Perc SI Screws	Stoppa & Lateral Window	Supine	Closed Reduction, DHS (NOF), ORIF (Shaft)	DHS + IM Nail	Lateral (DHS) + Retrograde (IM Nail)	
m, 69	Fall	NOF		ACPHT	Fix & Replace	Fix & Replace	Fix and Replace	Stoppa then KL	Supine then lateral	Fix & replace	THR (fix & replace)		DVT, wound dehiscen–e - washout required
m, 77	RTA	Proximal shaft		PW	–	Conservative	NA	NA	NA	Exfix, ORIF	IM Nail	Antegrade	
f, 86	RTA	Midshaft	LC II		Same Procedure, Femur	INFIX	INFIX	INFIX (Supra-Acetabular Entry)	Supine	ORIF	IM Nail (L)	Retrograde	
m, 26	RTA	Distal		PW	Femur	ORIF	Plate	KL	Lateral	ORIF	IM Nail + Poller Screw	Retrograde	
m, 32	RTA	Midshaft	LC I		Same Procedure, EUA P/A	EUA	NA	NA	NA	Exfix, ORIF	IM Nail	Retrograde	Femoral nonuni–n - revision required
m, 25	RTA	NOF	LC III		Femur	INFIX + SI Screws	INFIX + Perc SI Screws	Percutaneous + Infix	Supine	ORIF	DHS	Lateral	Deep infecti–n - washout required
m, 22	RTA	Proximal/proximal Shaft	LC I		Femur	EUA	NA	NA	NA	ORIF	IM Nail	Antegrade	
m, 17	Other	Proximal shaft		Tx	Femur	ORIF	Screws + Perc SI Screws	Stoppa & Lateral Window	Supine	ORIF	IM Nail	Antegrade	
f, 18	RTA	Midshaft	APC I		Femur	EUA	NA	NA	NA	ORIF	IM Nail	Antegrade	
m, 63	RTA	Midshaft	SIJ Disruption	TPW	Same Procedure, P/A	ORIF + Perc Fixation	Plate + Screws	KL	Lateral	ORIF	IM Nail	Retrograde	Hypertrophic ossification (grade 4)
f, 54	Fall from Height	NOF/ Intertrochanteric	LC I		–	Conservative	NA	NA	NA	ORIF	DHS	Lateral	Not recorded
m, 15	Fall	NOF		PW	Same Procedure, Femur	ORIF	Plate	KL	Lateral	ORIF	Cannulated Screws	KL Approach	Avascular necros–s - revision required
f, 86	Fall	NOF	LC I		–	Conservative	Hemiarthroplasty	Modified Hardinge	Lateral	Hemiarthroplasty	Hemiarthroplasty	Modified Hardinge approach, lateral position	Not recorded
m, 20	RTA	Proximal shaft	APC I		Same Procedure, P/A	ORIF	Plate + Screws	Stoppa	Supine	ORIF	IM nail	Retrograde	
f, 26	RTA	Subtrochanteric		PC	–	Conservative	NA	NA	NA	ORIF	IM nail	Antegrade	
m, 32	Fall from Height	Intertrochanteric	LC I		–	Conservative	NA	NA	NA	Closed reduction internal fixation	IM nail	Antegrade	Not recorded
m, 22	RTA	Proximal shaft	LC III		Femur	Exfix	Exfix	NA	NA	Exfix, ORIF	Proximal femoral locking plate	Lateral	
m, 24	RTA	Midshaft	APC I	Tx	Femur	ORIF	Plate + Screws	KL	Lateral	Closed reduction internal fixation	IM nail	Retrograde	
m, 39	Fall from Height	Proximal (trochanteric)	APC I		Same Procedure, P/A	ORIF	Plate	Stoppa	Supine	ORIF	Proximal femoral locking plate	Lateral	Incisional hern–a - elective repair required
m, 47	RTA	NOF	APC III		P/A	ORIF + Perc Fixation	Plate + Perc SI Screws	Stoppa (pubic symphysis) + lateral window (SI Joint)	Supine	ORIF	Cannulated screws	Lateral	Not recorded
m, 51	RTA	NOF + shaft	APC II		Same Procedure, P/A	ORIF + Perc Fixation	Plate + Screws + Perc SI Screw	Stoppa	Supine	ORIF	IM nail	Antegrade (IM Nail)	
m, 20	RTA	Midshaft		TPW	Femur	ORIF	Plate + Screws	KL (posterior column)	Lateral (posterior column)	ORIF	IM nail	Retrograde	
m, 20	RTA	Proximal shaft	APC I		Same Procedure, P/A	ORIF	Plate + Screws	Stoppa	Supine	ORIF	IM nail	Retrograde	
f, 26	RTA	Subtrochanteric		PC	–	Conservative	NA	NA	NA	ORIF	IM nail	Antegrade	
m, 32	Fall from Height	Intertrochanteric	LC I		–	Conservative	NA	NA	NA	Closed reduction internal fixation	IM nail	Antegrade	Not recorded
m, 22	RTA	Proximal shaft	LC III		Femur	Exfix	Exfix	NA	NA	Exfix, ORIF	Proximal femoral locking plate	Lateral	

M, male; f, female; NOF, neck of femur; RTA, road traffic accidents; EUA, examination under anaesthetic; ORIF, open reduction and internal fixation.

Of the 27 FH cases with surgically managed P/A fractures, 13 (48.1%) had femoral and P/A fractures managed in separate procedures, 11 (40.7%) had both fractures fixed during the same procedural window, and 3 (11.1%) were managed with an acute THR, wherein both fractures were fixed as part of the same procedure. Excluding the 3 acute THR cases, femoral fractures were fixed first in 17 (70.8%) of the remaining 24 cases (Table 3).

The mean (range) length of hospital stay for all FH cases was 28.3 (7–171) days. The weight-bearing status on discharge is detailed in Table 2. 29 patients had weight-bearing restrictions, with P/A fractures responsible for the weight-bearing status in 18 (62.1%) cases, femoral fractures were responsible for 4 (13.8%) cases, both fractures were equally responsible in 4 (13.8%) cases, and the remaining 3 (10.3%) weight-bearing status were due to other associated orthopaedic fractures.

### Complications and readmissions (of surgically managed cases)

Amongst the 27 surgically managed FH cases, 26 (96.3%) had available follow-up data, of a mean (range) of 11.9 (1–33) months. The total complication rate was 53.8% (*n* = 14), with 26.9% (*n* = 7) occurring within the same admission, and 34.6% (*n* = 9) occurring post discharge. The rate of readmission/revision surgeries was 34.6% (*n* = 9). The respective breakdowns are detailed in Table 4.

**Table 4.** Comparison of complication and readmission rates between “floating hip” cases and control groups.*

	Group		*p-*Value
	Cases (*n* = 26)	Controls (*n* = 25)	
	*n* (%)	*n* (%)	
Outcomes			
Post-op complications (total)	14 (53.8)	5 (20.0)	0.025
**Within same admission**	7 (26.9)	3 (12.0)	
Deep infections	2 (11.5)	0 (0.0)	
Pulmonary embolism (PE)/DVT	2 (3.8)	1 (4.0)	
Sciatic nerve injury	1 (3.8)	1 (4.0)	
Others	2 (7.7)	1 (4.0)	
** Post-discharge**	9 (34.6)	2 (10.0)	
Wound infections	1 (11.5)	1 (4.0)	
Femoral nonunion	4 (15.4)	NA	
Avascular necrosis (AVN)	1 (3.8)	0 (0.0)	
Heterotrophic ossification (Grade 4)	1 (3.8)	0 (0.0)	
Post-traumatic OA	0 (0.0)	1 (4.0)	
Others	2 (7.7)	0 (0.0)	
Required revision surgery/readmissions	9 (34.6)	3 (12.0)	0.06
Wound washout	2 (7.7)	2 (8.0)	
Revision THR	1 (3.8)	1 (4.0)	
Incisional hernia repair	1 (3.8)	0 (0.0)	
Revision of locking screws	1	0 (0.0)	
Femoral revision fixation	4 (15.4)	NA	

DVT, deep vein thrombosis; OA, osteoarthritis; THR, total hip replacement.

* Only patients who underwent surgical management and with available follow-up data were included in this analysis and formed the baseline.

The complication rate for Type A, B and C FH injuries was 37.5%, 50.0% and 66.7% respectively. An increased complication rate was not significantly associated with increased age (39.2 vs. 37.3 years, *p* = 0.82), time from admission to P/A fixation (3.90 days vs. 1.90 days, *p* *=* 0.10), or with choice of surgical management (52.9% in ORIF vs. 33.3% in acute THR, *p* = 0.431).

### Matched case control comparisons

The basic demographics, injury descriptions and fracture classifications of FH cases and controls are detailed in Table 2. As expected, there were no significant differences in the average age, sex ratio, average BMI, injury mechanisms, or fracture patterns between case and control groups (*p* > 0.05). FH cases had a significantly higher rate of associated orthopaedic injuries (81.0% vs. 54.8%, *p* *=* 0.01), but a similar rate of other non-orthopaedic injuries (73.8% vs. 69.1%).

Table 2 illustrates differences in management between FH cases and controls. There were minimal differences in the choice of surgical procedure, and in weight bearing status on discharge. FH cases, however, did require more total theatre admissions (mean of 2.5 vs. 1.19 admissions, *p* *<* 0.001), and have significantly longer hospital stays than controls (28.3 vs. 14.9 days, *p* = 0.02)

Of the 25 controls who underwent surgical management, all had available follow-up data. Table 4 compares the post-op complications and re-admission rates of FH cases versus controls. Compared to the controls, FH cases had a significantly higher rate of both post-op complications (53.8% vs. 20.0%, *p* = 0.025) and had a greater proportion of re-admissions/revision surgery, though this was not statistically significant (34.6% vs. 12.0%, *p* = 0.06).

## Discussion

The “floating hip” is a complex and severe injury pattern accompanied by high rates of complications and associated injuries. Our study provides a detailed description of the fracture patterns, injury mechanisms, management, and post-operative outcomes of FH injuries. In addition, our study demonstrates significant differences in the rate of other orthopaedic injuries, the length of hospital stay, number of OR admissions, and the rate of complications in FH cases compared to patients presenting with similar P/A injuries but without an ipsilateral femoral fracture.

Few studies have conducted a controlled comparisons between FH cases and controls.^5,16^ Zamora-Navas et al.^5^ attempted a form of comparison to patients without femoral involvement. Though the authors observed differences in values of complication rates and quality of life outcomes between the two groups, these were not statistically significant. In addition, the authors did not adjust or match their controls for any additional confounders. In contrast, Audretsch et al.^16^ found that FH injuries were operated on more frequently (62.8% vs. 39.1%, *p* *=* 0.003) than propensity matched cases, but found no significant difference in surgical outcomes. Our study builds upon their previous work, ensuring our control group was closely matched by age, gender, BMI, mechanism (energy) of injury, and fracture classification (all *p* > 0.05). Even after the implementation of rigorous matching criteria, however, we still observed significant differences between FH cases and controls.

Table 5 provides a brief summary of the relevant (study size ⩾ 10 patients) studies on the FH injury over the last 25 years. Our proportion of 30.9% Type A, 54.8% Type B and 14.3% Type C FH injuries largely matches the reported proportions previously cited in literature,^5,9,13^ with the exception of Burd et al.,^8^ who interestingly observed a larger proportion of Type C FH injuries than either Type A or B.

**Table 5.** Summary of prominent studies investigating “floating hip” injuries over the last 25 years.

Study name, year	Floating hip definition	Study numbers	Overall management and order of fixation	Complication and readmission rates	Comparisons to controls (if any)
Audretsch et al.^16^	P/A + femur fracture	43 patients 14 Type A 29 Type B	62.8% of P/A fractures required surgical treatment Femur fixed first in 70% of cases	8 cases of morbidity, 3 cases of mortality	Floating hip injuries operated on more frequently (62.8% vs. 39.1%, *p* = 0.003), but no difference in surgical outcomes
Brioschi et al.^17^	P/A + femur fracture	45 patients; 16 Type A 24 Type B 5 Type C	57.5% of P/A fractures required internal fixation 80% of femoral fractures required internal fixation Femur fixed first in 60% of cases	23 complications (51.1%) - 4 mortality - 4 amputation - 1 emboli - 10 local complications	No comparisons
Cech et al.^18^	P/A + femur fracture	69 patients; 52 type A 3 Type B 14 Type C	All femoral fractures fixed surgically All pelvic fractures fixed surgically 71% of acetabular fractures fixed surgically Order of fixation not explicitly mentioned	45 Complications (65%) - 11 thrombo-embolism - 8 peripheral nerve injuries - 8 heterotopic ossification (HO)	No comparisons
Meena et al.^19^	Acetabular + femur fracture	34 patients; all Type A	All femoral and acetabular fractures fixed surgically Femur fixed first in 100% of cases	12 Complications (35%) - 4 femoral nonunion - 4 infections - 1 heterotopic ossification - 1 sciatic nerve injury	No comparisons
Hammad et al.^20^	Acetabular + femur fracture	27 patients; all Type A	All femoral and acetabular fractures fixed surgically, same surgical setting, with femoral fractures fixed first	Overall complication rate not reported - 3 Infections - 4 arthritis - 2 femoral nonunion - 8 sciatic nerve injury - 5 heterotopic ossification	No comparisons
Liu et al.^12^	Acetabular + femur fracture	18 patients; all Type A	All femoral fractures fixed surgically 10/18 (55.6%) of acetabular fractures fixed surgically (ORIF) Femoral and Acetabular fractures fixed simultaneously via same incision/operation	9 Complications (50%) - 3 Post traumatic arthritis (Kellgren Lawrence grade ⩾2), 2 Grade 3 HO, 4 AVN 3 revisions/readmissions (16.7%) - 3 THA revision	No comparisons
Zamora-Navas et al.^5^	P/A + femur fracture	25 patients; 13 Type A 12 Type B	All femoral + P/A fractures surgically treated. Sequential treatment, in 100% of cases femoral fractures addressed first	10 complications (40%) - 7 HO, 2 neurological,1 PTO	No significant differences in EUROQOL (*p* > 0.05) Lower complication rate in controls (17.9%)
Cannada et al.^11^	Acetabular + femur fracture	101 patients; all Type A	All femoral + acetabular fractures surgically treated. 99% of patients underwent fixation of femur first, with 31% undergoing fixation within the same procedure.	48 major complications (47.5%) - 7 AVN, 29 HO, 8 deep infection, 4 PE 33 revisions/readmissions (32.7%)	No comparisons
Siavashi^21^	P/A + femur fracture	11 patients; 4 Type A 3 Type B 4 Type C	All femoral + P/A fractures surgically treated. 91% of cases were treated from proximal to distal (P&A fixation first before femoral fixation)	5 complications (45%) - 2 AVN - 2 femoral nonunion - 1 DVT 3 revisions/readmissions (27%) - 3 THA revision	No comparisons
Pavelka et al.^13^	P/A + femur fracture	54 patients; 15 Type A 28 Type B 7 Type C	All femoral + P/A fractures surgically treated. 83% of cases had femoral stabilisation before P/A fixation. Remaining cases had simultaneous treatment.	24 complications (44.4%) 10 early complications - 2 infections, 2 nerve injury, 6 imperfect reductions 14 late complications - 12 HO, 2 AVN	No comparisons
Burd et al.^8^	P/A + femur fracture	57 patients; 15 Type A 17 Type B 25 Type C	80% of acetabular & 55% of pelvic fractures surgically treated. 94% of femoral fractures surgically treated. Femoral fractures were always fixed at first operation.	36 complications (63%) - 7 DVT, 2 fem nonunion, 1 AVN fem head, 7 HO (>Grade 3), 19 sciatic nerve palsy	No comparisons
Liebergall et al.^3^	Acetabular + femur fracture	20 patients; all Type A	All femoral + acetabular fractures surgically treated. 65% treated in same procedure, 35% femoral fracture fixed first.	4 Complications (20%) - 1 PE, 1 displaced fracture, 2 PTO 3 revisions/readmissions (15%) - 3 THR (15%)	No comparisons
Müller et al.^9^	P/A + Femur Fracture	42 patients; 25 Type A 12 Type B 5 Type C	56.7% of acetabular fractures & 66.7 of pelvic fractures surgically treated. 95% of femoral fractures surgically treated. 38.5% of cases femur fixed first, 61.5% of cases P/A fracture fixed first.	14 complications (33.3%) - 3 DVT, 1 deep, 1 superficial infection, 1 ARDS, 4 sciatic nerve injury, 4 femoral nonunion 9 revisions/readmissions (21.4%) - 2 wound washout, 4 femoral revision, 3 acetabular refixation	No comparisons

P/A, pelvis or acetabulum; THA, total hip arthroplasty; AVN, avascular necrosis; DVT, deep vein thrombosis; PE, pulmonary embolism; PTO, post-traumatic osteoarthritis; ARDS, adult respiratory distress syndrome.

All femoral fractures within our FH series required surgical management. Such findings have been unanimously reported in almost all previous studies (Table 5). For P/A fractures however, surgical management was not always required - in most of such FH cases, the pelvic or acetabular fractures were often minimally displaced, deemed stable, or had little influence on the function of the hip joint during weight bearing. For 1 case of severe polytrauma in patient with significant comorbidities, the risks of P/A fixation were deemed to outweigh the benefits of fixation. Similar observations have again been corroborated in previous literature.^9,12^ Whenever surgical management of P/A fractures was deemed necessary, the femur was often fixed first, an order observed in most other case series as well. The beneficial effects of earlier fixation of femoral fractures are thought to stem from the increased physiological and mechanical stability; earlier stabilisation of the femur facilitates enables a stable platform for better positioning, exposure and reduction of P/A fractures, reduces the occurrence of significant bleeding, lowers fat emboli risks, and may also help patients achieve earlier mobilisation.^3,5,8,11,13^ Nonetheless, some studies have suggested pelvic fractures should be addressed first if the pelvis is a significant source of bleeding, and may potentially precipitate haemodynamic instability.^12,19,22^ Interestingly, Audretsch et al.^16^ and Wu et al.^22^ reported observing no significant differences in outcomes between a femur first versus pelvic/acetabular first strategy in managing FH cases.

While antegrade nailing remains the mainstay for femoral shaft fractures, debate remains whether this approach remains optimal in FH cases with concomitant acetabular fractures. Authors have cited concerns over increased potential of wound complications, and interference with proximal incisions during the Kocher-Langenbeck (KL) approach.^7,8,23^ Furthermore, tractional requirements with the antegrade approach may potentially predispose to secondary injury to unstable pelvic fractures or re-bleeding from the pelvic cavity.^22^ Within our case series, although the presence of a FH injury was not an absolute contraindication to an antegrade approach, this approach was generally only utilised during cases with either (1) stable/conservatively managed P/A fractures, (2) or in cases with proximal shaft/trochanteric involvement. The retrograde approach was often favoured in more unstable P/A injuries, whenever the KL approach was indicated, or with distal / midshaft femoral fractures. Nonetheless, Bishop et al.^7^ notably demonstrated no increased rate of wound-related complications in a case series of 16 FH patients treated with an antegrade approach, though they did note a potentially increased risk of heterotopic ossification.

The overall rate of complications and readmissions in our FH series was 50.0% and 26.9% respectively. These rates are similar to those of previous studies (Table 5), which range from 20–63.2% for complications, and 15–32.7% for readmissions accordingly. Combined P&A fractures often result in suboptimal outcomes,^24,25^ and it was unsurprising that a high rate of complications (66.7%) was observed in those with Type C FH injuries. Though age at injury is often associated with complication rates,^26,27^ this was not significant in our study. We suspect as FH injuries are often higher-energy fractures seen in younger populations, age may not be as crucial a factor in influencing post-surgical outcomes.

Femoral nonunions were the most common complication (15.4%) in our surgically managed patients. This rate is on the higher end of literature reported values, which range from 4.6% to 13.9%.^28–31^ We posit this may be due to an imposed weight-bearing status from the ipsilateral P/A fracture. Earlier weight-bearing is associated with better outcomes for femoral fractures, while P/A fractures often necessitate a period of non/protected weight-bearing.^32,33^ We suggest this is a factor that could possibly underlie the higher rates of femoral nonunion within our FH cases.

Strengths of this study include being the first to conduct comparisons with an extensively matched controlled group, and the high follow-up rates of both cases (96.3%) and controls (100%) that underwent surgical management. Furthermore, we report a relatively large collection of this rare injury presentation managed at a single major trauma centre under consistent surgical protocols. Nonetheless, this study does have some limitations. Firstly, our study was retrospective in design. Secondly, although we had a good follow-up rate with a median duration of 10 months (range 1–33 months), this duration may have been insufficient to robustly identify longer-term manifestations such as avascular necrosis (AVN), post-traumatic osteoarthritis (PTO), heterotopic ossification (HO), and/or conversion to THA. In addition, follow-up data were extracted from records of patient visits to post-op clinics, rather than prospectively collected as part of specific study protocol, and thus may not have been as robust. Furthermore, obtaining sufficient sample sizes to enable statistically significant conclusions is difficult for this uncommon injury, and precludes statistical adjustments for other potential confounding factors. The greater number of orthopedic injuries in FH cases for example, may potentially have affected outcomes considerably. Lastly, we did not manage to collect data on functional or patient reported outcomes, which could have added an additional dimension to our results.

## Conclusion

The “floating hip” injury often presents in young males suffering from high-energy fractures. These injuries often involve either the acetabulum (Type A) or pelvis (Type B), with concurrent pelvic and acetabular (Type C) fractures a rarer but often more severe presentation. Femoral fractures always required surgical management, and were often fixed first, while certain cases allowed for P/A fractures to be managed conservatively. After comparison to rigorously matched controls, significant differences were observed in the rate of other orthopaedic injuries, the length of hospital stay, and the rate of complications in FH cases. Clinicians should be aware of the associated complication profile to achieve optimal management strategies and patient outcomes.

## Supplemental Material

sj-pdf-1-hpi-10.1177_11207000231160075 – Supplemental material for The floating hip injury: a descriptive study and case-control analysis Click here for additional data file. Supplemental material, sj-pdf-1-hpi-10.1177_11207000231160075 for The floating hip injury: a descriptive study and case-control analysis by Mark Y Z Wong, Marios Ghobrial, Win M Han, Joseph Alsousou, Andrew Carrothers, Peter Hull, Daud Chou and Jaikirty Rawal in HIP International
