# Supplementary material for: The floating hip injury: a descriptive study and case-control analysis
Source: Hip Int. 2023 Mar 13;34(1):122–33. doi: 10.1177/11207000231160075 (PMC10787386; doi:10.1177/11207000231160075)
Supplement: sj-pdf-1-hpi-10.1177_11207000231160075 – Supplemental material for The floating hip injury: a descriptive study and case-control analysis [file sj-pdf-1-hpi-10.1177_11207000231160075.pdf]

APPENDIX

Supplementary Table 1: Distribution of Acetabular and Pelvic Fracture Patterns in Floating Hip cases and controls

	**Cases (N = 42)**	**Controls (N = 42)**
**<u>Pelvic Fractures</u>**		
APC I	6	6
APC II	3	4
APC III	4	2
LC I	10	11
LC II	2	1
LC III	3	2
**Total Patients (n)**	**29**	**27**
**<u>Acetabular Fractures</u>**		
Elementary		
Anterior Wall	1	1
Anterior Column	2	1
PW	3	4
PC	1	2
Tx	3	1
Associated		
ABC	2	2
TPW	3	2
T-Shaped	1	1
ACPHT	2	3
**Total Patients (n)**	**19**	**16**

Supplementary Table 2: Full list of other orthopaedic & non-orthopaedic injuries in Floating Hip cases

Floating Hip Case	Other orthopaedic injuries	Other non-orthopaedic injuries
FH01	#R Distal Radius, #R Clavicle, #L Thumb	TBI, #Rib, Pneumothorax
FH02	#Left Humerus, #left Ulna, #Right Calcaneus and Cuboid	Multiple Facial #, Traumatic Ruptured Bladder, Bilateral Pneumothorax
FH03	R tibial/fib #, MM, Right clavicle #, right leg compartment syndrome	Head Injury, Skull Frac, Right Pneumothorax, Rhabdomyolysis
FH04	R Patella, R scapular spine, C7, left distal ulna fracture	Facial fractures, Rib fractures, Pulmonary contusions, Liver laceration.
FH05	L calcaneal, 1st MT base, 2nd, 3rd, 4th and 5th MT neck fractures, Left Achilles tendon laceration	Head Laceration, Left flail chest segment, right rib fractures
FH06	Ipsilateral NOF fracture	NIL
FH07	NIL	NIL
FH08	NIL	TBI, Parietal Haematoma, Splenic Haematoma
FH09	R Metatarsal & R Ankle Fracture	TBI, Lung contusions
FH10	NIL	Haematoma / Abrasion to Bilateral Iliac Regions
FH11	# Right Clavicle Undisplaced, # Transverse Process L5	NIL
FH12	L Calcaneal - Subtalar joint #	NIL
FH13	NIL	Severe TBI & Facial & SB Fractures, Thoracic Injuries, Rib Fractures, Pneumothorax,
FH14	Shoulder Dislocation, Foot 5th MT Head #	NIL
FH15	Open R hand R distal radius Open R index finger	TBI? Complex Facial #
FH16	# T5 Transverse Process	NIL
FH17	NIL	NIL
FH18	R Open Ankle # dislocation	NIL
FH19	#R Radius & Scaphoid, #T12	NIL
FH20	Open L Tibial Wounds, #L1,2&5 Transverse Process	NIL
FH21	Nil	NIL
FH22	R Clavicle #, Open Elbow # dislocation + degloving, open L humerus #, R thumb injury	TBI, Bilat HTX & PTX, Traumatic Bowel Injury
FH23	R Periprosthetic Knee #,	Head Laceration, BL Renal Lacerations, R Flail Chest
FH24	Right Ankle Avulsion #	NIL

FH25	R Open Humeral #, T12-L5 Right TP #, T7 Spinous process #	R 12th Rib #, Retroperitoneal Haematoma
FH26	R #tib & fib #R olecranon R 5th PIPJ dislocation	Haemopneumothorax, Liver Laceration, Scrotal Laceration, Rib Fractures, Pericardial Effusion, Terminal Ileum Perforation
FH27	Communited # of scapula & L Clavicle, Unstable T2-T3 #,	TBI, Severe Chest & Abdominal Trauma, Fractured Rib Fragments, BL Haemopneumothorax, Bilateral Pulmonary Contusions, Splenic Lacerations, Kidney Lacerations, Haematoma
FH28	Displaced L Humeral #, L2 Transverse Process #	L 6th Rib #, L Lung Contusions with Small L PTX, Colonic Contusion,
FH29	R Patella Tendon Rupture, #T1 & T2 Transverse Process	L upper Rib #, Pulmonary Contusions, Anterior Mediastinal Haematoma, Facial Lacerations, Nasal Bone #, Displaced Bladder
FH30	Hip Dislocation, R Patella #, L Olecranon #, R Distal tib/fib #	Sternal #, R PTX, Split Tooth, BL Rib #
FH31	#R wrist, #R Fibula Head, #C7 Wedge	R Maxillary #, R facial laceration, TSAH (Traumatic Subarachnoid Haemorrhage)
FH32	NIL	NIL
FH33	NIL	NIL
FH34	#BL Radius & Ulna	L Upper Lobe Contusion, Retroperitoneal Haematoma, Compression on L Distal Ureter
FH35	R Tibia # (Tibial Plateau)	NIL
FH36	NIL	Traumatic Subarachnoid
FH37	#L1 & L5 TP	Tension PTX, Abdo Compartment Syndrome, Transection of R internal iliac, Cardiac arrest
FH38	BL Radial #	Aortic Arch Transection, Mediastinal Haematoma, Pleural Effusion, Ptx
FH39	#L1, L Shoulder Supraspinatus Tear	NIL
FH40	#L Fibular Neck, L Knee PCL Injury, Lumbar Plexopathy	L PTX, Low GCS, Rhabdomyolysis
FH41	#R Forearm, #L3 L4 TP,	NIL
FH42	#R Clavicle, #R Olecranon & Proximal Ulna, #L Distal Radius, #R Patella, R Proximal tibial Wound, R Subtalar Dislocation, L Foot toe dislocations, #C7,	#Occipital
